# Reported Changes in Eating Habits Related to Less Healthy Foods and Beverages during the COVID-19 Pandemic among US Adults

**DOI:** 10.3390/nu14030526

**Published:** 2022-01-26

**Authors:** Sohyun Park, Seung Hee Lee, Amy L. Yaroch, Heidi M. Blanck

**Affiliations:** 1 Division of Nutrition, Physical Activity, and Obesity, National Center for Chronic Disease Prevention and Health Promotion, Centers for Disease Control and Prevention, 4770 Buford Highway NE, Atlanta, GA 30341, USA; xde5@cdc.gov (S.H.L.); hcb3@cdc.gov (H.M.B.); 2 Gretchen Swanson Center for Nutrition, 14301 FNB Parkway, Suite 100, Omaha, NE 68154, USA; ayaroch@centerfornutrition.org

**Keywords:** added sugars, unhealthy snacks, desserts, sugar-sweetened beverage, COVID-19 pandemic, US adults, diet

## Abstract

Background: The COVID-19 pandemic has triggered stress, anxiety, and disruption to many individuals’ daily lives, which might impact eating habits. Objective: To examine changes in eating habits related to less healthy foods and beverages during the early phase of the COVID-19 pandemic among US adults. Design: Cross-sectional study. Participants/setting: Authors used SummerStyles data gathered in June 2020 among 3916 US adults (≥18 years). Main outcome measures: The outcome of interest was the reported frequency of consuming more (1) unhealthy snacks and desserts including chips, cookies, and ice cream and (2) sugar-sweetened beverages (SSBs) like regular soda, fruit drinks, sports/energy drinks, sweetened coffee/teas during the COVID-19 pandemic. Responses were categorized as Never/Rarely, Sometimes, or Often/Always. Explanatory variables were sociodemographics, weight status, and census regions. Statistical analyses performed: We used multinomial regressions to calculate adjusted odds ratios (AOR) for Sometimes or Often/Always consuming more unhealthy snacks/desserts (vs. Never/Rarely); and Sometimes or Often/Always more SSBs (vs. Never/Rarely). Results: Overall, 36% of adults reported sometimes consuming more unhealthy snacks/desserts; 16% did so often/always. Twenty-two percent of adults reported sometimes drinking more SSBs; 10% did so often/always. Factors significantly associated with higher odds of reporting often/always consuming more unhealthy snacks/desserts were younger adults (AOR range = 1.51–2.86 vs. adults ≥65 years), females (AOR = 1.58 vs. males), non-Hispanic Black (AOR = 1.89 vs. non-Hispanic White), lower household income (AOR = 2.01 for <USD 35,000 vs. ≥USD 100,000), and obesity (AOR = 1.56 vs. underweight/healthy weight). Factors significantly associated with odds of Often/Always drinking more SSBs were being younger (AOR range = 2.26–4.39 vs. adults ≥65 years), non-Hispanic Black (AOR = 3.25 vs. non-Hispanic White), Hispanic (AOR = 1.75 vs. non-Hispanic White), non-Hispanic Other race/ethnicity (AOR = 2.41 vs. non-Hispanic White), lower education (AOR = 2.03 for ≤high school; AOR = 1.80 for some college vs. college graduate), lower household income (AOR range = 1.64–3.15 vs. ≥USD 100,000), and obesity (AOR = 1.61 vs. underweight/healthy weight). Conclusions: Consuming more sugary foods and SSBs during the first phase of the pandemic was higher in younger adults, lower-income adults, people of racial/ethnic minority groups, and adults with obesity. Dietary shifts to less healthy foods and drinks may influence metabolic health if sustained long-term. Implementing strategies to support individual’s healthy eating habits during the ongoing pandemic and the pandemic recovery may benefit health and wellness.

## 1. Introduction

The consumption of unhealthy food and beverage (or junk food) is high among adults in the United States [1,2]. The 2015–2018 National Health and Nutrition Examination Survey (NHANES) revealed that US adults (≥19 years) consumed 47% of their total energy (or 1043 kcal/day) from junk foods, and 75% of their total sugar intake came from junk foods [1]. Among US adults, of the total sugar intake (54.4 g/day) derived from junk foods, 41% was from sugar-sweetened beverages (SSBs), 23% was from desserts, and 9% was from confectionery [1]. Additionally, an earlier NHANES (2015–2016) showed that US adults (≥20 years) consumed about 16 teaspoons of added sugars on a given day [3]. SSBs are the top sources of added sugars [4]. A study reported that 63% of US adults drank SSBs at least once per day in 2010 and 2015 [5]. High intake of ultra-processed foods, including junk foods, is a public health concern because greater intake of ultra-processed foods is associated with adverse health risks in adults including higher risk for developing cardiovascular disease, obesity, and metabolic syndrome, and for all-cause mortality [6,7,8].

The SARS-CoV-2 coronavirus 2019 (COVID-19) pandemic has caused psychological stress, anxiety, and disruption to many individuals’ daily lives due to various reasons including safer-at-home orders, pandemic-induced unemployment/income loss, disruption to childcare, school campus and retail closures, and concerns about contracting COVID-19 [9,10,11]. A previous study showed that 14% of US adults (*n* = 1468) stated symptoms of serious psychological distress in April 2020, which is about a three-fold increase from 2018 (4%) [12]. Stress influences eating behaviors, which can result in undereating or overeating [13,14,15]. For instance, acute stress can cause loss of appetite resulting in undereating, while chronic stress might be associated with an increased preference for palatable foods that are high in sugar and fat [14]. A few studies have examined the relationship between COVID-19 stress and food intake among US adults [16,17]. A cross-sectional study among US adults (≥18 years) reported that a March 2020 cohort (*n* = 868) consumed about 14% more added sugars than the February 2019 cohort (*n* = 247) [16]. Furthermore, higher stress attributable to the COVID-19 pandemic was related to more eating to cope and a higher intake of added sugars [16]. Another US study conducted among adults (*n* = 428) during the months of May and June 2020 found that the highest self-reported food intake frequency was sweets/desserts—17.4 times per week during COVID-19 [17]. The same study also showed that emotional overeating alone, as well as COVID-19 stress and emotional overeating combined had significant effects on increased frequency of consuming sweets/desserts [17]. Shortages of food products during the COVID-19 pandemic have changed food distribution and trade [18], which might have impacted people’s eating behaviors (e.g., changes in access to different foods at home or changes in the cost or availability of foods).

Some studies have examined eating behavior variations among adults during the COVID-19 pandemic, but most were conducted in other countries [19,20,21,22]. While a few studies were conducted in the United States, these studies had small sample sizes, and due to their methods, study findings cannot be generalized to the whole US adult populations [16,17,23,24]. Thus, we examined changes in the consumption of SSBs and less healthy snacks and desserts during the early phase of the COVID-19 pandemic among US adults using the 2020 SummerStyles survey.

## 2. Methods

### 2.1. Study Sample and Survey Administration

A cross-sectional study was conducted using data from the 2020 SummerStyles survey, which is an online survey led by Porter Novelli Public Service [25]. These data were collected through an online market research panel (i.e., Ipsos’ Knowledge Panel). This online panel was intended to be representative of the noninstitutionalized US population. Panel members were recruited randomly by mail through probability, address-based sampling. The survey collected a wide variety of information on health-related knowledge, attitudes, behaviors, and conditions around important public health topics. Survey participants received points (worth about USD 5) for participating in the panel, which could be used to redeem cash and prizes. If needed, families were provided with a device (i.e., laptop or tablet) and access to the Internet. Personal identifiers were excluded in the dataset provided to the Centers for Disease Control and Prevention (CDC); thus, the current study was exempt from the CDC institutional review board. 

**Figure 1:**
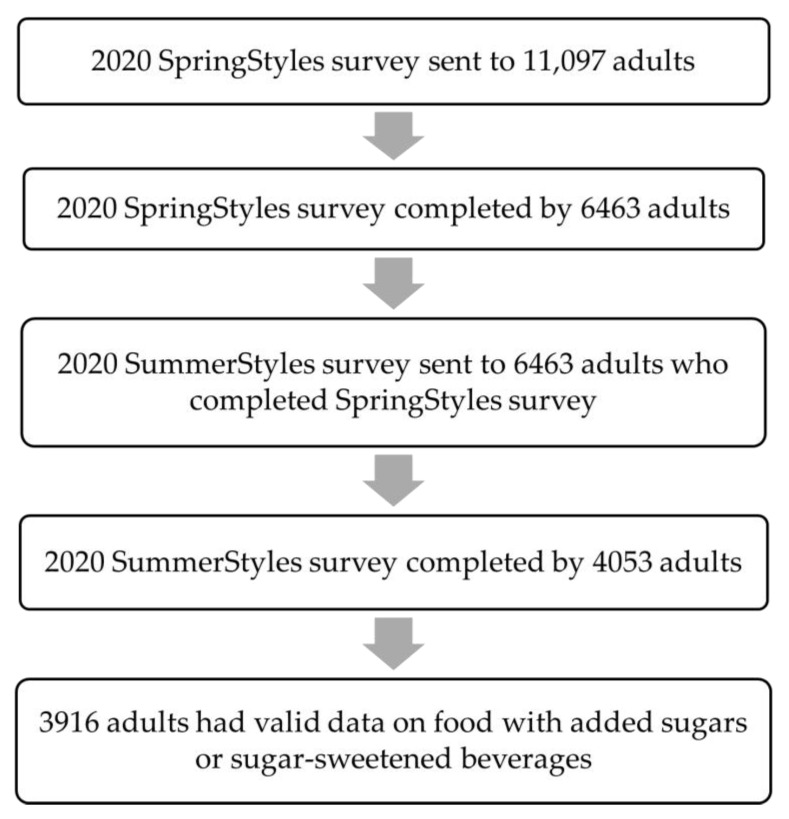
Analytic sample flow chart for SummerStyles survey, 2020.

The SummerStyles survey was directed at individuals who participated in the SpringStyles survey (an initial wave). In March–April 2020, the SpringStyles survey was sent to 11,097 panelists aged ≥18 years and 6463 adults completed the survey (Figure 1), which yielded a 58.2% response rate. During June 2020, the SummerStyles survey was sent to 6463 adults who had completed the SpringStyles survey. The SummerStyles survey was completed by 4053 adults aged ≥18 years (62.7% response rate). To match the Current Population Survey proportions, the survey data were weighted according to age, sex, race/ethnicity, education, household income, household size, US census region, metropolitan status, and parental status of children aged 12–17 years old. Of 4053 adults who completed the SummerStyles survey, 137 (4.3%) adults with missing or invalid data for outcome variables (i.e., foods with added sugars or SSBs) were excluded from the current analysis, leaving an analytic sample of 3916 adults. Of note, the outcome variables were only asked about in the SummerStyles survey and not in the SpringStyles survey.

### 2.2. Outcome Variables 

The outcome variables were the reported frequency of consuming more unhealthy snacks and desserts and drinking more SSBs and they were assessed using the following questions: “Think about any changes that may have occurred with your food or eating patterns after the COVID-19 pandemic, after 11 March 2020. How often did you and your household do the following?” Two statements were analyzed from this question: (1) “Consumed more unhealthy snacks and desserts including chips, cookies, and ice cream” and (2) “Drank more sugary drinks like regular soda, fruit drinks, sports or energy drinks, sweetened coffee/teas drinks”. Respondents selected one response per statement using a Likert scale. For this analysis, the response options were combined into Never/Rarely, Sometimes, and Often/Always. These survey items were created by authors who are experts in this research field including nutritional and/or behavioral scientists. 

### 2.3. Explanatory Variables

The explanatory variables included sociodemographic characteristics, weight status, and census regions. The sociodemographic variables were age groups (18–24, 25–44, 45–64, or ≥65 years), sex, race/ethnicity (non-Hispanic White, non-Hispanic Black, Hispanic, or non-Hispanic Other race/ethnicity), education level (≤high school, some college, or college graduate), marital status (married/domestic partnership or not married), annual household income (<USD 35,000, USD 35,000–USD 74,999, USD 75,000–USD 99,999, or ≥USD 100,000), and currently having child(ren) aged <18 years. Weight status was grouped into 3 types according to the self-reported height and weight data of participants, which were used to compute body mass index (BMI): underweight/healthy weight (BMI < 25 kg/m^2^), overweight (BMI 25 to < 30 kg/m^2^), and obesity (BMI ≥ 30 kg/m^2^) [26]. We combined underweight and healthy weight into a sole category because only 2% of adults were underweight. The census regions of residence were Northeast, Midwest, South, or West [27].

### 2.4. Statistical Analysis 

For unadjusted bivariate analyses, descriptive statistics were used to examine the characteristics of those who, after the start of the COVID-19 pandemic (1) consumed more unhealthy snacks and desserts, and (2) drank more SSBs. χ^2^ tests were used to examine the association between their eating habits and sociodemographic characteristics, weight status, and census regions. A *p* value of ≤0.05 was used to define statistical significance.

For adjusted analyses, multinominal logistic regression analysis was used to estimate the adjusted odds ratios (AORs) and 95% confidence intervals (CIs) for the association between (1) consuming more unhealthy snacks and desserts, and (2) drinking more SSBs and variables related to sociodemographic characteristics, weight status, and census region. Each model included all variables in one model, and the reference outcome category was Never/Rarely. Of the 3916 adults with outcome data, the sample size was reduced to 3841 adults for logistic regression models due to missing weight status (*n* = 69) and currently having children aged <18 years (*n* = 6). All statistical analyses were performed with the Statistical Analysis Software (SAS) Version 9.4 (SAS Institute Inc., Cary, NC, USA) using survey procedures to account for the sampling weights.

## 3. Results

Overall, 35.7% of adults reported sometimes consuming more unhealthy snacks/desserts, while 16% did so often/always during the early phase of the COVID-19 pandemic (Table 1). Based on bivariate analyses, consuming more unhealthy snacks/desserts during this time was significantly related to all characteristics except census region (χ^2^ tests, *p* < 0.05; Table 1). For example, the proportion of adults who reported often/always consuming more unhealthy snacks/desserts was highest among adults aged 25–44 years, female, non-Hispanic Black adults, those with ≤high school education, not married adults, those with an annual household income <USD 35,000, those with children aged <18 years, and those with obesity. Based on the multinominal logistic regression model using Never/Rarely as a reference group, factors significantly associated with higher odds of reporting often/always consuming more unhealthy snacks/desserts were younger adults (AOR range = 1.51–2.86 vs. adults ≥65 years), females (AOR = 1.58), non-Hispanic Black (AOR = 1.89 vs. non-Hispanic White), lower annual household income (AOR = 2.01 for <USD 35,000 vs. ≥USD 100,000), and obesity (AOR = 1.56 vs. underweight/healthy weight) (Table 1).

Overall, 21.6% of adults reported sometimes drinking more SSBs, while 9.6% did so often/always during the timeframe. Based on bivariate analyses, drinking more SSBs during the COVID-19 pandemic was significantly related to all characteristics except sex (χ^2^ tests, *p* < 0.05; Table 2). For example, the proportion of adults who reported often/always drinking more SSBs was highest among were adults aged 25–44 years, non-Hispanic Black adults, those with ≤high school education, not married adults, those with an annual household income <USD 35,000, those with children aged <18 years, those with obesity, and those living in the Midwest. Based on the multinominal logistic regression model using never/rarely as a reference group, factors significantly associated with odds of often/always drinking more SSBs were younger adults (AOR range = 2.26–4.39 vs. adults ≥65 years), non-Hispanic Black (AOR = 3.25), Hispanic (AOR = 1.75), non-Hispanic Other race/ethnicity (AOR = 2.41 vs. non-Hispanic White), lower education (AOR = 2.03 for ≤ high school; AOR = 1.80 for some college vs. college graduate), lower annual household income (AOR range = 1.64–3.15 vs. ≥USD 100,000), and obesity (AOR = 1.61 vs. underweight/healthy weight) (Table 2). 

## 4. Discussion

In the present study, about 1 in 2 adults reported sometimes or often/always consuming more unhealthy snacks and desserts during the early phase (March to June 2020) of the COVID-19 pandemic. Furthermore, about 1 in 3 adults reported sometimes or often/always drinking more SSBs during this spring and summer 2020 time period. Our findings aligned with previous studies conducted in the United States [17] and European countries [19,20] that showed increased self-reported consumption of unhealthy snacks, sugary foods/desserts, and SSBs during the COVID-19 lockdown period. For example, a study conducted in US adults (*n* = 428) during May and June 2020 found that 41% of adults reported increased consumption of sweets/desserts during the COVID-19 pandemic [17]. Another study conducted in adults living in Los Angeles (LA) County (*n* = 1080) during April and July 2020 found that 25% of adults reported consuming less healthy foods (e.g., less fruits and vegetables, and/or more sugary or fried food) during the COVID-19 pandemic [23]. Increased consumption of unhealthy snacks, sugary foods/desserts, and SSBs during the COVID-19 pandemic could be in part due to psychological stress triggered by the COVID-19 pandemic. A previous study found that increased COVID-related stress was significantly associated with higher motivation for sweet snacks and to preferring fast food, and among 429 US adults, respondents were willing to pay more for these foods compared to other food types [28]. Additionally, perceived stress during the COVID-19 pandemic was significantly related to emotional eating [29,30] and motives for food choices [29].

In the present study, younger adults experienced increased consumption of both unhealthy snacks/desserts and SSBs compared to older adults (≥65 years). Consistent with our findings, a previous study reported that younger adults (18–30 years) had 2.4 times higher odds of reporting unhealthy dietary change during the COVID-19 pandemic than older adults (≥65 years) in LA County [23]. Another study reported that older adults had significantly less emotional eating from COVID-19 related worries and psychological distress than young adults [20]. It is possible that older adults might have experienced less stress associated with COVID-19 pandemic, such as disruption of childcare or pandemic-induced unemployment/income loss or have more habitual eating habits that are developed and sustained over time. 

We found that women had increased odds of consuming more unhealthy snacks and desserts during the early phase of the COVID-19 pandemic than men. A previous study conducted in Norway found that female participants had a significantly higher prevalence of emotional eating compared to males [20]. However, another study reported that associations between COVID-19 stress and eating to cope were stronger among men than women [16]. There were no sex differences in consuming more SSBs in the present study. Regarding unhealthy snacks and desserts, non-Hispanic Black adults in the present study had significantly higher odds for consuming more compared to non-Hispanic White adults. For SSBs, non-Hispanic Black, Hispanic and non-Hispanic Other racial/ethnic adults had significantly higher odds for drinking more SSBs than non-Hispanic White adults. Somewhat different from our findings, the LA County study reported that non-Hispanic Asian adults and mixed race had significantly higher odds for unhealthy dietary change during the COVID-19 pandemic than non-Hispanic White adults [23]. It is difficult to compare our study findings with the LA County study because of the geographical specificity of the LA County. Nevertheless, the negative effects of the COVID-19 pandemic have a greater impact on people of certain minority racial/ethnic groups as previous studies showed that COVID-19 cases, hospitalization, and deaths are higher among Black and Latino/Hispanic populations than White populations in the United States [31,32]. Stress is undoubtedly more pronounced in people of color during the COVID-19 pandemic, and the disproportionate stress might occur partially because of existing inequities experienced at the population level [33]. 

We found that consuming more unhealthy snacks and desserts as well as SSBs during the early phases of COVID-19 pandemic significantly differed by household income, weight status, and having minors in the home. Our study found adults with lower annual household income (<USD 35,000) had significantly higher odds for consuming more unhealthy snacks/desserts and SSBs than those with higher annual household income. Inconsistent with our findings, poverty level was not significantly associated with unhealthy dietary change among adults in LA County [23]. The COVID-19 pandemic might have worsened food insecurity among low-income households that were struggling with food insecurity prior to the COVID-19 pandemic, partially because of the economic impacts of the COVID-19 pandemic such as fewer resources, less flexibility in their jobs, and/or decreasing household income [34]. In our study, adults with obesity had significantly higher odds for consuming more unhealthy snacks/desserts and SSBs than those with underweight or healthy weight. Consistent with our results, the LA County study reported that adults who were diagnosed with obesity had significantly higher odds of unhealthy dietary changes [23]. A longitudinal study of young adults found that those who have experienced weight stigma had more depressive symptoms and used eating as a survival strategy during the COVID-19 pandemic [35]. We found that adults with children (<18 years) had higher odds for sometimes consuming more unhealthy snacks/desserts and SSBs. Similar to our study findings, the LA County study found that households with children reported increased odds of unhealthy dietary change [23]. This could be in part because adults with children might have experienced more disruptions in their daily life and stress because of virtual schooling or childcare closures during the COVID-19 pandemic, and unhealthy dietary changes may be a coping mechanism. Another possibility might be that more unhealthy foods and beverages could have been in the home and accessible to parents because children were home and requesting those items.

Our findings on the consumption of more unhealthy snacks/desserts and SSBs during the COVID-19 pandemic can help provide information on groups possibly in need of extra support during the ongoing pandemic. In addition, this type of study can help inform future pandemics or other public health emergencies that may cause stress or other disruptions and increase dietary shifts. Unhealthy dietary changes, particularly if sustained, could increase the risk of increased adiposity and the development or worsening of obesity, cardiovascular disease, type 2 diabetes, and lung disease, which increases the risk for complications of COVID-19 [36]. The strengths of the current study include a large sample size, assessing factors in June 2020 (temporally close to the lockdown time due to the pandemic), and a relatively diverse sample with regards to race/ethnicity. 

### Limitations

Despite these strengths, the study has several limitations. First, we used data from a cross-sectional survey (i.e., a SummerStyles survey); thus, causational associations cannot be established. Second, the data are subject to bias such as recall or social desirability bias, because the data are self-reported. Third, the study did not measure actual changes in unhealthy snacks and desserts and SSBs intake nor did it assess decreases in consumption. Fourth, the definition of unhealthy snacks could vary widely among respondents because of the way the unhealthy snack question was asked. Fifth, the validity and reliability of the survey items (i.e., outcome variables) were not tested. Finally, the study findings may not be generalized to the whole US adult population, because the initial sample was selected from individuals willing to be part of the larger online panel. However, the data were weighted to be comparable with Current Population Survey proportions.

## 5. Conclusions

In conclusion, changes in eating habits were common, with about 1 in 2 adults reporting consuming more unhealthy snacks and desserts and about 1 in 3 adults reporting drinking more SSBs sometimes or often/always during the early phase of the COVID-19 pandemic. Consuming more of these less healthy foods and beverages was higher in younger adults, people of racial/ethnic minority groups, lower-income adults, and adults with obesity. Dietary changes to less healthy foods/drinks may impact metabolic health if sustained in the long-term. Supporting healthful eating habits of individuals during the pandemic and the pandemic recovery is important. Implementing strategies (e.g., social marketing, education, ensuring food and nutrition security) to ensure these dietary changes are not permanent may benefit health and wellness. 

## Figures and Tables

**Table 1:** Characteristics of respondents and their associations with consuming more unhealthy snacks and desserts including chips, cookies, and ice cream after the COVID-19 pandemic among US adults, SummerStyles Survey, 2020.

Characteristic	All Respondents % ^a^	After the COVID-19 Pandemic, 11 March, 2020, How Often Did You and Your Household Consume More Unhealthy Snacks and Desserts including Chips, Cookies, and Ice Cream				
		Bivariate Analysis ^b^			Multinomial Analysis ^c^	
		Never/Rarely % ^a^ ± SE	Sometimes % ^a^ ± SE	Often/Always % ^a^ ± SE	Sometimes AOR (95% CI)	Often/Always AOR (95% CI)
**Total (*n* = 3916) ^d^**	100	48.3 ± 1.0	35.7 ± 0.9	16.0 ± 0.8	-	-
Age						
18–24 years	10.6	**42.8 ± 4.9**	**37.0 ± 4.8**	**20.2 ± 3.9**	1.43 (0.90, 2.27)	**2.58 (1.41, 4.70)**
25–44 years	34.5	**43.1 ± 1.6**	**35.2 ± 1.6**	**21.6 ± 1.4**	1.23 (0.97, 1.58)	**2.86 (2.01, 4.06)**
45–64 years	33.2	**50.2 ± 1.4**	**36.7 ± 1.4**	**13.1 ± 1.0**	1.12 (0.91, 1.37)	**1.51 (1.11, 2.05)**
≥65 years	21.7	**56.4 ± 1.7**	**34.1 ± 1.6**	**9.5 ± 1.0**	Reference	Reference
Sex						
Male	48.6	**52.2 ± 1.4**	**34.4 ± 1.3**	**13.4 ± 1.0**	Reference	Reference
Female	51.4	**44.6 ± 1.3**	**36.9 ± 1.3**	**18.5 ± 1.1**	**1.25 (1.05, 1.49)**	**1.58 (1.23, 2.02)**
Race/ethnicity						
White, non-Hispanic	64.2	**51.4 ± 1.1**	**34.4 ± 1.1**	**14.2 ± 0.8**	Reference	Reference
Black, non-Hispanic	11.5	**40.0 ± 3.1**	**35.3 ± 3.0**	**24.8 ± 3.0**	1.18 (0.86, 1.62)	**1.89 (1.26, 2.82)**
Hispanic	15.8	**43.1 ± 2.9**	**39.2 ± 2.9**	**17.6 ± 2.2**	1.32 (1.00, 1.75)	1.38 (0.96, 1.99)
Other, non-Hispanic	8.5	**45.9 ± 3.5**	**39.2 ± 3.5**	**15.0 ± 2.6**	**1.50 (1.07, 2.10)**	1.35 (0.84, 2.16)
Education level						
High school or less	38.1	**45.0 ± 1.7**	**36.8 ± 1.7**	**18.2 ± 1.4**	1.19 (0.94, 1.50)	1.20 (0.87, 1.65)
Some college	27.9	**47.0 ± 1.9**	**37.2 ± 1.8**	**15.8 ± 1.5**	1.21 (0.97, 1.51)	1.17 (0.86, 1.60)
College graduate	34.1	**53.1 ± 1.4**	**33.2 ± 1.3**	**13.7 ± 1.0**	Reference	Reference
Marital status						
Married/domestic partnership	62.1	**50.4 ± 1.1**	**35.5 ± 1.1**	**14.0 ± 0.8**	Reference	Reference
Not married	37.9	**44.9 ± 1.8**	**35.9 ± 1.8**	**19.2 ± 1.5**	1.04 (0.85, 1.28)	1.16 (0.86, 1.55)
Annual household income						
<USD 35,000	20.5	**37.2 ± 2.3**	**39.3 ± 2.4**	**23.5 ± 2.1**	1.31 (0.97, 1.77)	**2.01 (1.35, 2.99)**
USD 35,000–USD 74,999	26.6	**49.0 ± 1.9**	**34.9 ± 1.8**	**15.2 ± 1.4**	0.96 (0.76, 1.21)	1.09 (0.78, 1.51)
USD 75,000–USD 99,999	14.0	**50.6 ± 2.7**	**33.0 ± 2.4**	**16.5 ± 2.2**	0.89 (0.68, 1.16)	1.21 (0.82, 1.79)
≥USD 100,000	38.9	**52.3 ± 1.4**	**35.2 ± 1.4**	**12.4 ± 1.0**	Reference	Reference
Currently have children aged <18 years (*n* = 3910)						
Yes	27.5	**41.5 ± 1.7**	**38.4 ± 1.7**	**20.0 ± 1.5**	**1.32 (1.07, 1.63)**	1.22 (0.90, 1.66)
No	72.5	**50.9 ± 1.2**	**34.6 ± 1.1**	**14.5 ± 0.9**	Reference	Reference
Weight status ^e^ (*n* = 3847)						
Underweight/healthy weight	35.6	**52.0 ± 1.8**	**32.0 ± 1.6**	**16.1 ± 1.4**	Reference	Reference
Overweight	32.0	**51.4 ± 1.7**	**35.3 ± 1.6**	**13.3 ± 1.2**	1.21 (0.97, 1.50)	0.97 (0.71, 1.32)
Obesity	32.4	**41.2 ± 1.6**	**40.0 ± 1.6**	**18.7 ± 1.3**	**1.64 (1.32, 2.04)**	**1.56 (1.17, 2.09)**
Census regions of residence						
Northeast	17.8	46.1 ± 2.3	36.9 ± 2.2	16.9 ± 1.9	1.10 (0.86, 1.41)	1.26 (0.89, 1.79)
Midwest	20.9	47.7 ± 2.1	34.2 ± 1.9	18.1 ± 1.7	0.90 (0.71, 1.15)	1.10 (0.80, 1.52)
South	37.7	47.3 ± 1.6	36.7 ± 1.6	16.0 ± 1.2	Reference	Reference
West	23.6	52.2 ± 2.0	34.3 ± 1.9	13.5 ± 1.4	0.83 (0.66, 1.05)	0.81 (0.58, 1.13)

Abbreviations: SE: standard error; AOR: adjusted odds ratio; 95% CI: 95% confidence intervals. ^a^ Weighted percent may not add up to 100% because of rounding. ^b^ χ^2^ tests were used for each variable to examine differences across categories. Variables with *p* < 0.05 were bolded. ^c^ All variables listed in Table 1 were included in one multinomial logistic regression model and based on a sample of 3841 adults without missing data. Reference outcome category was Never/Rarely. Significant findings are bolded based on the 95% confidence intervals (i.e., the confidence interval does not include 1). ^d^ Unweighted sample size. ^e^ Based on calculated body mass index (BMI) (kg/m^2^): underweight/healthy weight, BMI < 25; overweight, BMI 25 to <30; obesity, BMI ≥ 30.

**Table 2:** Associations between characteristics of respondents and drinking more sugar-sweetened beverages (SSBs) among US adults after the COVID-19 pandemic, SummerStyles Survey, 2020.

Characteristic	After the COVID-19 Pandemic, 11 March, 2020, How Often Did You and Your Household Drink More SSBs like Regular Soda, Fruit Drinks, Sports or Energy Drinks, Sweetened Coffee/Teas Drinks				
	Bivariate Analysis ^a^			Multinomial Analysis ^b^	
	Never/Rarely % ^c^ ± SE	Sometimes % ^c^ ± SE	Often/Always % ^c^ ± SE	Sometimes AOR (95% CI)	Often/Always AOR (95% CI)
Total (*n* = 3916) ^d^	68.8 ± 1.0	21.6 ± 0.9	9.6 ± 0.6	-	-
Age					
18–24 years	**58.6 ± 4.9**	**30.8 ± 4.6**	**10.6 ± 3.1**	**2.10 (1.26, 3.49)**	**3.00 (1.34, 6.71)**
25–44 years	**62.8 ± 1.6**	**23.4 ± 1.4**	**13.8 ± 1.2**	**1.56 (1.15, 2.10)**	**4.39 (2.69, 7.19)**
45–64 years	**72.3 ± 1.3**	**19.1 ± 1.2**	**8.5 ± 0.9**	1.09 (0.84, 1.41)	**2.26 (1.45, 3.51)**
≥ 65 years	**78.0 ± 1.6**	**18.0 ± 1.5**	**4.0 ± 0.7**	Reference	Reference
Sex					
Male	70.5 ± 1.3	20.8 ± 1.2	8.7 ± 0.8	Reference	Reference
Female	67.2 ± 1.4	22.3 ± 1.2	10.4 ± 0.9	1.12 (0.90, 1.38)	1.18 (0.87, 1.60)
Race/ethnicity					
White, non-Hispanic	**74.8 ± 1.0**	**18.1 ± 0.9**	**7.1 ± 0.6**	Reference	Reference
Black, non-Hispanic	**52.1 ± 3.2**	**28.3 ± 2.8**	**19.6 ± 2.8**	**2.06 (1.49, 2.85)**	**3.25 (2.02, 5.22)**
Hispanic	**57.1 ± 2.9**	**31.3 ± 2.8**	**11.7 ± 1.9**	**1.83 (1.35, 2.49)**	**1.75 (1.14, 2.67)**
Other, non-Hispanic	**67.9 ± 3.3**	**20.8 ± 2.9**	**11.2 ± 2.3**	**1.53 (1.03, 2.27)**	**2.41 (1.42, 4.11)**
Education level					
High school or less	**59.6 ± 1.8**	**27.9 ± 1.7**	**12.4 ± 1.2**	**1.89 (1.44, 2.48)**	**2.03 (1.32, 3.12)**
Some college	**68.4 ± 1.8**	**21.1 ± 1.6**	**10.4 ± 1.3**	**1.40 (1.06, 1.84)**	**1.80 (1.22, 2.67)**
College graduate	**79.4 ± 1.2**	**14.9 ± 1.0**	**5.8 ± 0.7**	Reference	Reference
Marital status					
Married/domestic partnership	**72.8 ± 1.0**	**19.2 ± 0.9**	**8.0 ± 0.7**	Reference	Reference
Not married	**62.3 ± 1.8**	**25.5 ± 1.7**	**12.2 ± 1.2**	1.11 (0.87, 1.41)	1.12 (0.77, 1.62)
Annual household income					
<USD 35,000	**51.9 ± 2.5**	**31.4 ± 2.4**	**16.7 ± 1.9**	**1.84 (1.31, 2.59)**	**3.15 (1.93, 5.14)**
USD 35,000–USD 74,999	**68.2 ± 1.8**	**20.9 ± 1.5**	**10.3 ± 1.2**	1.08 (0.81, 1.45)	**1.64 (1.07, 2.51)**
USD 75,000–USD 99,999	**66.8 ± 2.7**	**22.8 ± 2.4**	**10.4 ± 1.9**	1.30 (0.93, 1.81)	**2.02 (1.25, 3.25)**
≥USD 100,000	**78.4 ± 1.3**	**16.5 ± 1.2**	**5.1 ± 0.7**	Reference	Reference
Currently have children aged <18 years (*n* = 3910)					
Yes	**62.8 ± 1.7**	**24.4 ± 1.5**	**12.8 ± 1.3**	**1.33 (1.03, 1.72)**	1.21 (0.82, 1.78)
No	**71.0 ± 1.4**	**20.6 ± 1.0**	**8.4 ± 0.7**	Reference	Reference
Weight status ^e^ (*n* = 3847)					
Underweight/healthy weight	**72.5 ± 1.7**	**18.5 ± 1.5**	**9.0 ± 1.1**	Reference	Reference
Overweight	**71.6 ± 1.7**	**21.6 ± 1.5**	**6.9 ± 1.0**	1.30 (0.99, 1.71)	0.85 (0.56, 1.27)
Obesity	**61.8 ± 1.7**	**25.4 ± 1.5**	**12.8 ± 1.2**	**1.64 (1.26, 2.15)**	**1.61 (1.11, 2.35)**
Census regions of residence					
Northeast	**73.1 ± 2.2**	**18.5 ± 1.9**	**8.4 ± 1.4**	0.86 (0.62, 1.18)	0.95 (0.60, 1.50)
Midwest	**69.3 ± 2.0**	**19.1 ± 1.6**	**11.7 ± 1.6**	0.86 (0.64, 1.15)	1.13 (0.76, 1.69)
South	**66.7 ± 1.6**	**23.0 ± 1.5**	**10.3 ± 1.0**	Reference	Reference
West	**68.6 ± 2.0**	**23.9 ± 1.8**	**7.5 ± 1.1**	1.06 (0.80, 1.40)	0.76 (0.51, 1.15)

Abbreviations: SSBs: sugar-sweetened beverages; SE: standard error; AOR: adjusted odds ratio; 95% CI: 95% confidence intervals. ^a^ χ^2^ tests were used for each variable to examine differences across categories. Variables with *p* < 0.05 are bolded. ^b^ All variables listed in Table 2 were included in one multinomial logistic regression model and based on a sample of 3841 adults without missing data. Reference outcome category was Never/Rarely. Significant findings are bolded based on the 95% confidence intervals (i.e., the confidence interval does not include 1). ^c^ Weighted percent may not add up to 100% because of rounding. ^d^ Unweighted sample size. ^e^ Based on calculated body mass index (BMI) (kg/m^2^): underweight/healthy weight, BMI < 25; overweight, BMI 25 to <30; obesity, BMI ≥ 30.

## Data Availability

Data sharing is not applicable to this article.
